# Lethality of Honey Bee Stings to Heavily Armored Hornets

**DOI:** 10.3390/biology10060484

**Published:** 2021-05-29

**Authors:** Gaoying Gu, Yichuan Meng, Ken Tan, Shihao Dong, James C. Nieh

**Affiliations:** 1 CAS Key Laboratory of Tropical Forest Ecology, Xishuangbanna Tropical Botanical Garden, Chinese Academy of Sciences, Kunming 650000, China; gugaoying@xtbg.ac.cn (G.G.); mengyichuan@xtbg.ac.cn (Y.M.); kentan@xtbg.ac.cn (K.T.); 2 University of Chinese Academy of Sciences, Beijing 100049, China; 3 Center of Plant Ecology, Core Botanical Gardens, Chinese Academy of Sciences, Mengla 666303, China; 4 Division of Biological Sciences, Section of Ecology, Behavior, and Evolution, University of California San Diego, La Jolla, CA 92093, USA

**Keywords:** arms race, hornet predation, honey bees, defense

## Abstract

**Simple Summary:**

The co-evolution of attack and defense strategies between *Apis* and *Vespa* is a good model for studying arms races. Some honey bee species and subspecies can kill hornets with heat balls that generate heat and carbon dioxide. However, the role of stinging as a defense against hornets has been discounted, even though stings and venom are important honey bee weapons. No studies, to date, have tested the role of bee sting venom alone or in conjunction with elevated temperature on hornet survival. We found that bees can sting hornets but most hornets (87%) are able to remove bee stings less than 1 min after being stung, perhaps explaining why stinging is not considered a major anti-hornet defense. However, we show that such bee stings can kill hornets and demon-strate that the combination of sting venom and being heated is the most lethal to hornets.

**Abstract:**

The heat ball defense of honey bees against their sympatric hornet predators is a classic and spectacular outcome of a co-evolutionary race. Hundreds of bees can encapsulate a hornet within a large ball that kills it with elevated heat. However, the role of stinging in this defense has been discounted, even though sting venom is an important weapon in bees. Surprisingly, no studies have tested the role of bee sting venom alone or in conjunction with elevated temperature on hornet survival. We surveyed dead *Vespa velutina* hornets found near and inside *Apis cerana* colonies and found stings retained in hornet bodies, most often in an intersegmental neck-like region, the veracervix. Experimentally stinging hornets in this region with *A. cerana* and *Apis mellifera* guards significantly increased hornet mortality. The combination of sting venom and elevated heat ball temperature (44 °C) was the most lethal, although there was no synergistic interaction between sting venom and temperature. As expected, hornet mortality increased when they were stung more often. The average amount of venom per insect species and the length of stinger lancets correlated with insect mass. Sting venom thus remains important in the arms race between bees and their hornet predators.

## 1. Introduction

The co-evolutionary arms race between predators and prey has led to remarkable innovations in behaviors and structures. A consistent theme is that evolutionary innovations are often not eliminated when new elements arise, but instead are incorporated within a multi-element strategy [1]. The social insects provide classic examples because their colonies are tempting spatial concentrations of resources and they have therefore evolved defense strategies that combine individual weaponry and armor with the advantages of coordinated behaviors. For example, multiple honey bee species serve as prey for *Vespa* hornets [2] because small flying insects, typically pollinators, are an important food source for wasp larvae [3]. The group defense abilities of honey bees have led to an interesting phenomenon, heat-balling, in which honey bees can defend their colonies by encasing an attacking hornet in a ball of defenders. Heat-balling is a classic co-evolutionary example because it typically occurs and is effective between sympatric predators and their prey: *Apis cerana japonica* and *Vespa simillima xanthoptera* or *Vespa mandarinia* [4], *Apis cerana cerana* and *Vespa velutina* or *V. mandarinia* [5], *A. mellifera cypria* and *Vespa orientalis* [6], and *A. mellifera ligustica* and *Vespa crabro* [7]. In contrast, *A. mellifera ligustica* ineffectively heat-balls the allopatric *V. velutina* [8], and therefore suffers in areas where this hornet species has invaded [9].

Attention has been focused on the heat generated in the ball, a spectacular aspect of this defense [10]. Ono et al. [11] reported that >500 *A. cerana japonica* workers can quickly engulf the giant hornet, *V. mandarinia japonica*, generating an average temperature of 47 °C inside the ball. Similarly, *A. cerana japonica* can form a 46.1 °C ball around *Vespa simillima xanthoptera* [12] and *A. mellifera* can form a 44.0 ± 0.96 °C heat ball around *Vespa simillima xanthoptera* [13]. Tan et al. (2005) reported maximum internal ball temperatures of 45.3–45.6 °C for *A. cerana cerana* heat-balling *Vespa velutina* [14]. Likewise, *A. mellifera ligustica* can reach temperatures of 39.9 °C to 44.3 °C when heat-balling its sympatric predator, *V. crabro* [7].

However, these elevated temperatures may not solely account for hornet deaths because heat balls do not always achieve lethal temperatures for hornets. The lethal temperature for *V. crabro* is 44.2 ± 0.5 °C, but the mean inner temperature of the bee balls is 39.9 ± 7.4 °C [7]. In other cases, the lethal temperature more closely matches the heat ball temperature. Tan et al. (2005) placed *V. velutina* in incubators and showed that the lethal thermal limit is 45.7 ± 0.5 °C, close to the maximum temperature of 45.6 ± 0.1 °C in an *A. cerana* heat ball [14]. *Apis cerana japonica* workers could generate heat balls with an internal temperature of 47 °C for 20 min around *V. mandarinia* [11]. However, research shows that *V. mandarinia* can survive more than 10 min in an incubator at this temperature, and therefore CO_2_ generated within the heat ball is a major factor in its lethality [15,16]. Bees can also asphyxiate the hornet by blocking its respiratory spiracles [6].

Interestingly, the role of bee sting venom has been discounted as part of the heat ball defense, although sting venom plays a key role in other honey bee defenses. Ono et al. (1987) found no *A. cerana japonica* stings in the corpses of heat-balled *V. simillima xanthoptera* and noted that the honey bees forming the ball did not sting even when placed in the palm of one’s hand [12]. In contrast, *A. mellifera* workers do sting hornets, and two to three stings were found in intersegmental membranes of *V. simillima xanthoptera* [12]. Baracchi et al. (2010) observed that, in some cases, *A. mellifera* left stings inside the corpses of *V. crabro* [7].

In prior experiments with tethered *V. velutina*, we observed *A. cerana cerana* [5] and *A. mellifera ligustica* [8] guards trying to sting the hornet predator, particularly during the initial stage of heat-balling [8]. Both *A. cerana* and *A. mellifera* use sting venom volatiles as an alarm pheromone, and gas chromatography analyses revealed that *A. cerana* and *A. mellifera* released sting alarm pheromones during heat-balling [8]. Ono et al. [12] used the presence of bee stings remaining in the hornet to determine if they were stung. However, the barbed lancets of bees’ stings do not always remain in the victim. From our preliminary observations, we found that most bees could withdraw their stings after stinging a hornet, perhaps because they could not sufficiently penetrate the hornet cuticle to engage the sting barbs. A bee sting also left a very small hole in the hornet cuticle, making it difficult to discern a stinging event from visual examination alone if no sting was retained. In addition, hornets can likely sting bees when being heat-balled, although this is also not well understood. The role of stinging by bees and hornets in heat-balling, therefore, remains relatively unexplored. We hypothesized that sting venom can contribute to the death of hornets and that the combination of stinging and elevated temperature is more lethal than either alone.

## 2. Materials and Methods

We conducted our experiments in an apiary with *A. cerana cerana* and *A. mellifera ligustica* colonies at the Southwest Asia Biodiversity Research Institute in Kunming, China in September of 2019, a time when both bee species were actively foraging and naturally preyed upon by hornets. At this site, there were more than 10 hornet colonies in trees, on roofs, and on building eaves. We used 20 *A. cerana* colonies and 20 *A. mellifera* colonies, each consisting of four combs housed inside a wooden box, whose health was verified by weekly inspections consisting of carefully opening the colonies, checking both sides of each comb for evidence of parasites and disease, and then replacing the combs. Live *V. velutina* hornets were captured at beehive entrances with an insect net while they were hunting foraging bees. We used a different hornet per trial. Sample sizes for all three experiments are shown in Table 1 and Table 2.

### 2.1. Experiment 1: Effects of Honey Bee Stings on Hornet Mortality

**Figure 1:**
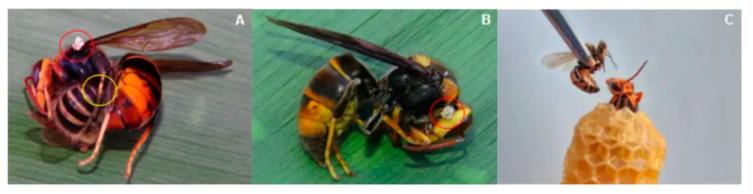
(**A**) An *A. cerana* guard attempting to sting (yellow circle) a *V. velutina* worker that has already been naturally stung in its thorax by another *A. cerana* (red circle). (**B**) An *A. mellifera* sting left in the veracervix of a fresh dead hornet (red circle). (**C**) A hornet restrained in wax that is about to be experimentally stung by an *A. mellifera* guard.

We monitored the nest entrances of 20 *A. cerana* colonies and 20 *A. mellifera* colonies over seven days from 10:00 to 15:00. We observed that 28 *V. velutina* foragers were heat-balled at the entrance of *A. cerana* colonies, but only two *V. velutina* foragers were heat-balled by *A. mellifera* colonies. Out of these 30 heat-balled hornets, only ten had honey bee stings in their bodies. One hornet had two stings in its veracervix, an intersegmental “neck-like” region [17], five had one sting each in the veracervix, one had a sting in its head capsule, two hornets were each stung once in their thoraces, and one was stung in a metathoracic leg (Figure 1). Through close observation, we found that most hornets (87%) were able to remove bee stings less than 1 min after being stung, so the majority of stings being left in the veracervix suggests that this structure may be particularly vulnerable.

To test the effects of honey bee stings on hornet mortality, we wrapped a freshly captured and healthy *V. velutina* forager with beeswax foundation, leaving its head and veracervix exposed (Figure 1c). We conducted this experiment at ambient air temperature at a site 5 m away from the honey bee colonies. For this experiment, we used bees from three *A. mellifera* colonies and three *A. cerana* colonies. We then caught a healthy guard bee with an aspirator, held the guard between forceps, and positioned it so that it stung the hornet’s veracervix. Because most hornets could remove bee stings less than 1 min after being stung under natural conditions (see above), we left the bee sting in the hornet for 30 s to ensure a standard exposure time. We tested the effects of 1, 2, or 3 stings by *A. mellifera* and *A. cerana* guards on hornet mortality measured over 3 h, choosing this maximum time interval because preliminary trials showed that hornets surviving beyond 3 h appeared to suffer no mortality. When hornets were multiply stung, each successive sting was administered with a 10 s interval between stings. We defined hornet death as the hornet becoming motionless and moribund, even when stimulated with forceps. Sham sting controls consisted of hornets that were identically restrained. They were not stung by bees. Instead, each hornet had its veracervix pierced to a depth of less than 1 mm with a fine, sterile needle. These sham sting controls were also observed for 3 h.

### 2.2. Experiment 2: Testing If Heat Accelerated Hornet Death

Both *A. cerana* and *A. mellifera* can heat-ball *V. velutina*, although the balls produced by *A. mellifera* are much smaller and less effective [14]. To determine if the heat generated in a ball can increase the deadliness of sting venom, we tested the effects of heat separately and in conjunction with sting venom. Previously, it was shown that the lethal temperature for *V. velutina* was 45.7 ± 0.48 °C and that the inner temperature of balls can sharply increase to 44 °C, which can be maintained for approximately 30 min in both *A. cerana* and *A. mellifera* [14]. We tested three groups of *V. velutina* hornets: sham stung by a needle (as above), stung by an *A. cerana* guard, or stung by an *A. mellifera* guard. Hornets were placed either at ambient (25 °C) or heated (44 °C) temperatures inside an incubator (BPN-150CH-UV) for 3 h, resulting in six different treatments. For this experiment, we used three *A. mellifera* colonies and three *A. cerana* colonies.

### 2.3. Experiment 3: Measuring Body Mass, Venom Mass, and Sting Length

We used an analytical balance (model ES1205A, Shanghai, China, accuracy of 0.01 mg) to measure insect body mass. To measure the amount of venom injected by each of the bee species and *V. velutina*, we calculated the amount of mass lost by the stinging insect after it was stung. To obtain accurate measurements, we first anesthetized the insect with CO_2_ (flow rate at 166 mL/s through 0.8 cm diameter tubing) until it ceased moving (5–7 s). The individual was then placed in a cage until it had recovered from the anesthesia and showed normal movement. Honey bees were given a hornet to sting as described above. Hornets were given a honey bee to sting (half of the hornets stung *A. mellifera* and the other half stung *A. cerana* guards). After stinging, the stinging insect (with its stinger still attached to its body) was carefully removed from the victim and weighed after re-anesthesia. Although honey bees often leave a detached stinger in the victim, we were careful to ensure that this did not happen when they stung the hornet. Thus, no honey bee bodily fluids were lost apart from the venom injected into the victim. The hornet sting is not barbed and is not left in the victim. We therefore attributed the loss of mass to the mass of the venom expelled by the stinging individual. As a control to account for potential mass changes, we took a different set of insects, applied the same before and after anesthesia treatment, waited 30 s between weighings, and calculated their average weight change. We also dissected out the sting apparatus from bees of both species and measured the lancet length with an optical microscope (Phenix, XSP-36-1600X). For this experiment, we used bees from three *A. mellifera* colonies and three *A. cerana* colonies.

### 2.4. Statistics

We used JMP Pro v14.2.0 (SAS Institute, Inc., Cary, NC, USA) statistical software. To analyze survival, we used Cox Proportional Hazards models and tested for model effects (all fixed) with Wald chi-square tests. To further explore significant differences within effects, we ran survival analyses and report the log-rank chi-square test results (applying the post-hoc Dunn–Sidak correction as appropriate and denoting significant results as DS). We used Analysis of Variance (ANOVA) to determine the effect of insect species on venom mass (log transformed), sting lancet length, and total fresh body mass (log transformed). We applied Tukey Honestly Significant Difference (HSD) tests to make corrected pairwise comparisons. For our mass control trials, we used Wilcoxon signed rank 2-tailed tests to determine if the mean mass difference (mass before−mass after) was significantly different from zero.

### 2.5. Data Accessibility Statement

All data are available at http://doi.org/10.5281/zenodo.4782609 (accessed on 23 May 2021).

## 3. Results

### 3.1. Experiment 1: Bee Stings Significantly Decreased Hornet Survival

**Figure 2:**
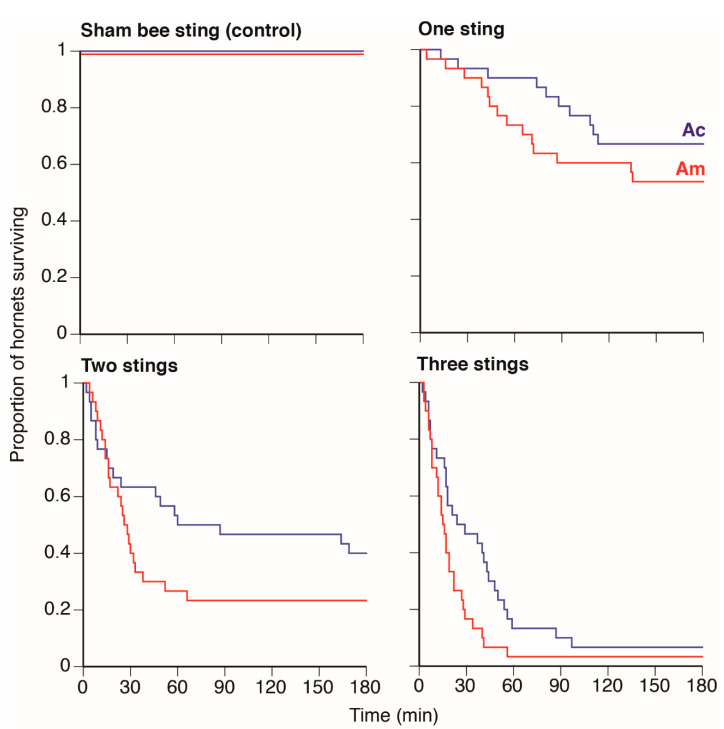
Effects of different numbers of bee stings from *A. cerana* (Ac) or *A. mellifera* guards (Am) on hornet survival over time. In the sham bee sting treatment, hornets were randomly assigned *a priori* as Ac or Am sham treatments and all survived (colored lines are slightly offset to facilitate visualization).

Hornet survival decreased as the number of bee stings increased, and *A. mellifera* stings generally resulted in lower hornet survival compared to *A. cerana* stings (sting type, Figure 2). There were significant effects of the number of stings received (Wald chi-square = 35.01, 3 d.f., *p* < 0.0001), sting type (Wald chi-square = 262.74, 1 d.f., *p* < 0.0001), and interaction of the number of stings × sting type (Wald chi-square = 235.37, 2 d.f., *p* < 0.0001). As expected, survival was lower when hornets received more stings. None of the bee stings were retained by the hornets. None of the hornets receiving the sham sting procedure died during any of the 3 h trials.

### 3.2. Experiment 2: Heat-Ball Temperature and Stinging Significantly Decreased Hornet Survival

**Figure 3:**
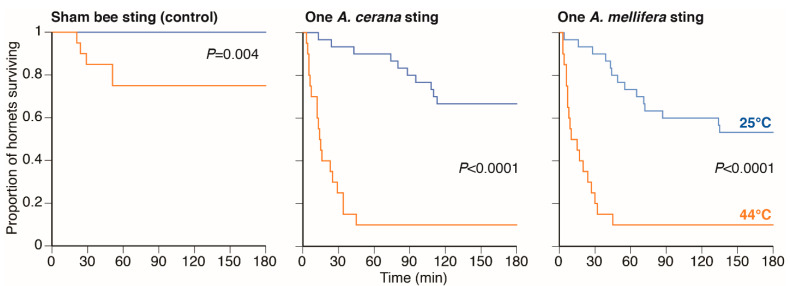
Effect of heat-balling temperature (=44 °C) and the species of bee sting upon hornet survival. The *p*-values derive from log-rank chi-square tests.

Heat (Wald chi-square = 54.52, 1 d.f., *p* < 0.0001^DS^) and sting type (sham sting, *A. cerana* sting, or *A. mellifera* sting: Wald chi-square = 32.27, 2 d.f., *p* < 0.0001^DS^) both significantly decreased hornet survival (Figure 3). However, there was no significant interaction of heat × sting type (Wald chi-square = 0.52, 2 d.f., *p* = 0.77), and thus no synergistic effect of heat and sting type. Increased temperature alone significantly reduced hornet survival by 25% at 3 h (log-rank chi-square = 8.33, 1 d.f., *p* = 0.004 ^DS^).

The combination of heat ball temperature (44 °C) and one bee sting significantly reduced survival by 57% (*A. cerana* sting: log-rank chi-square = 33.08, 1 d.f., *p* < 0.0001^DS^) and 53% (*A. mellifera* sting: log-rank chi-square = 25.88, 1 d.f., *p* < 0.0001^DS^) as compared to being stung by each respective species and maintained at ambient temperature (25 °C). When combined with elevated temperatures, *A. cerana* and *A. mellifera* stings decreased hornet survival approximately equally (Figure 3). None of the hornets receiving the sham sting and maintained at ambient temperature died during any of the 3 h trials.

### 3.3. Experiment 3: Venom Quantity and Sting Length Corresponded with Insect Body Mass

**Figure 4:**
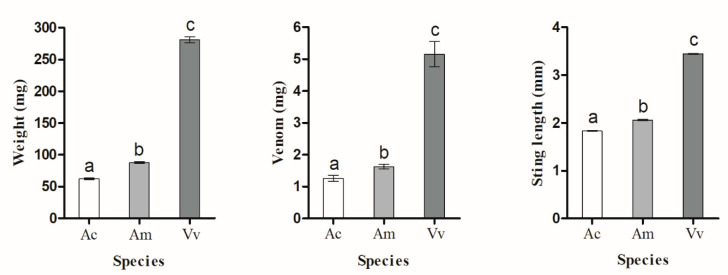
Comparisons between body mass, venom, and stinger length between *A. cerana* (Ac), *A. mellifera* (Ac), and *V. velutina* (Vv). Plots show the mean ± 1 standard error. Different letters indicate significant differences (Tukey HSD tests, *p* < 0.05).

Hornets produced more venom than *A. mellifera*, which in turn produced more venom than *A. cerana* (F_2,57_ = 131.82, *p* < 0.0001; all pairwise differences significant, Tukey HSD test, *p* < 0.05). Similarly, hornets had longer sting lancets than *A. mellifera*, which had longer lancets than *A. cerana* (F_2,57_ = 660.00, *p* < 0.0001; all pairwise differences significant, Tukey HSD test, *p* < 0.05). These differences matched the insect body masses (F_2,57_ = 131.82, *p* < 0.0001), which was also significantly different between all species (Tukey HSD test, *p* < 0.05, Figure 4). The percentage of venom mass to body mass was 1.8% (*V. velutina*), 1.8% (*A. mellifera*), and 2% (*A. cerana*).

As a control, we tested if the insects could have changed their mass during the 30 s between weighings or due to the CO_2_ anesthesia. The average mass differences (before − after mass) were 0.01 mg (*A. cerana*), 0.01 mg (*A. mellifera*), and 0.05 mg (*V. velutina*). They were not significantly different from a mean difference of zero for *A. cerana* (Wilcoxon signed-rank 2-tailed test, W = −3.0, *p* = 0.98), *A. mellifera* (Wilcoxon signed-rank 2-tailed test, W = 40.5, *p* = 0.17), or *V. velutina* (Wilcoxon signed-rank 2-tailed test, W = 45.0, *p* = 0.18). Thus, the weighing and anesthesia did not result in measurable weight changes.

## 4. Discussion

Heat-balling is an ingenious defense that has co-evolved to protect multiple honey bee species from hornet predators. It is known that high temperature, increased concentration of CO_2_, and blockage of the hornet’s respiratory system contribute to hornet death, but no detailed studies have focused on the effects of bee sting venom on hornet mortality. We show that both heating and sting venom are important and that hornets evidently possess an Achilles heel, their neck-like veracervix. Dead hornets found in and outside of *A. cerana* and *A. mellifera* bee colonies were stung in multiple locations, but most frequently in the veracervix. Our experiments showed that hornet survival significantly decreased as the number of stings in the veracervix increased, regardless of the stinging bee species (Figure 2). As expected, temperature also played a key role because hornets heated to 44 °C, the average heat ball temperature, had 57% reduced survival compared to being stung and maintained at ambient air temperature (21 °C). Heating alone only reduced survival by 25% after 3 h. At 44 °C, the median time to hornet death was 14.5 min for hornets stung by *A. cerana* and 12.5 min for hornets stung by *A. mellifera*. Our sham stinging treatment, on its own and at ambient temperature, did not increase hornet mortality over the trial duration. Finally, none of the hornets stung in the veracervix retained the bee stings, and thus simply censusing the number of stings retained on dead hornets likely underestimates the role of bee stinging.

As expected, the amount of venom per insect and the length of stinger lancets was closely related to insect mass. The significantly lower quantity of venom in an *A. cerana* as compared to an *A. mellifera* guard may account for the lower survival rate of hornets stung by *A. mellifera* as compared to *A. cerana* (Figure 2).

Honey bee venom contains multiple toxic compounds [18], and we expected that venom and heat shock would synergistically increase hornet mortality. For example, elevated temperatures could promote the spread of venom through the hornet’s body. However, we found no synergistic effect of these two factors, perhaps because heat degrades some venom components. Baracchi et al. (2010) reported another potentially lethal sting-related mechanism: hornets being covered with bee venom and alarm pheromone [7]. The effects of venom applied to the exoskeleton of the hornet, and whether it can enter the hornet’s spiracles or mouthparts, and thereby contribute to hornet death, would be fascinating to test. In addition, the precise contributions of elevated CO_2_, increased temperature, spiracular suffocation, and stinging have yet to be elucidated. Finally, the heat ball creates a physical constraint, reducing the mobility of the hornet and perhaps making it easier for bees to sting the hornet’s veracervix. The potential for synergy between these different possible mechanisms is interesting and deserves exploration.

The evolution of heat-balling and the problems suffered by bee species without a strong heat-balling defense against hornet predators [9] demonstrate that sting venom alone is not sufficient. One can therefore view heat-balling as a combination of offensive tactics that mutually achieve the same aim, particularly when colonies do not have a large number of defenders. Weaker colonies with fewer guards are far more vulnerable to hornet predation [19]. For larger hornets such as the giant hornet (*V. mandarinia*) even more defending bees are required. *Apis cerana* form larger balls (6-fold more bees) around the giant hornet (*V. mandarinia*) as compared to a smaller hornet (*V. velutina*) [5]. However, colonies with limited defenders could augment their defense with the additional toxicity of bee venom. Thus, old weapons still work and can provide an advantage in the knife’s edge balance of an arms race between predator and prey.

## 5. Conclusions

The present study demonstrates that honey bee stings can kill a hornet predator by stinging the neck-like veracervix. Furthermore, the combination of stinging and the high temperature generated by heat-balling also contributes to hornet death. This study provides the first evidence that honey bee stings are a key aspect of bee defense against hornets.

## Figures and Tables

**Table 1:** Sample sizes for experiment 1 and experiment 2 (in number of individuals).

Experiment	No. of *V. velutina*	Honey Bee Colony	No. of *A. cerana*	No. of *A. mellifera*
1	210	1	20	20
		2	20	20
		3	20	20
2	120	1	13	13
		2	14	14
		3	13	13

**Table 2:** Sample sizes for experiment 3 (in number of individuals). Data on body mass, venom mass, and sting length are shown in Figure 4.

Species	Body Mass	Venom	Sting Length
*V. velutina*	60	20	20
*cerana*	60	20	20
*mellifera*	60	20	20

## Data Availability

All data freely accessible, upon publication, http://doi.org/10.5281/zenodo.4782609.
